# Emergence of deadly viral haemorrhagic fever disease outbreaks in West Africa

**DOI:** 10.1080/21505594.2023.2176980

**Published:** 2023-02-14

**Authors:** Widaliz Vega-Rodriguez, Hinh Ly

**Affiliations:** Department of Veterinary & Biomedical Sciences, College of Veterinary Medicine, University of Minnesota, Twin Cities, St Paul, MN, USA

**Keywords:** Ebola virus, Marburg virus, Lassa virus, viral disease outbreaks, West Africa, viral hemorrhagic fevers

## Abstract

Recent viral hemorrhagic fever (VHF) disease outbreaks caused by Ebola virus (EBOV) and Marburg virus (MARV) in West Africa are unique and alarming. The intents of this editorial are to highlight what is known about these viruses and the disease outbreaks that they cause in the African continent and elsewhere and to raise awareness of a related virus called Lassa virus (LASV) that causes endemic viral hemorrhagic fever infections and frequent outbreaks in West Africa.

*Filoviridae* family includes three genera: *Cuevavirus, Marburgvirus (MARV), and Ebolavirus (EBOV)* [1]. The genus *Cuevavirus* includes the species Lloviu cuevavirus with a single known virus member called Lloviu virus (LLOV) that is distantly related to MARV and EBOV but is taxonomically similar to them in terms of having similar filamentous virion and linear non-segmented, negative single-stranded RNA genome structures. Unlike LLOV, which is not known to infect humans but has been found to be associated with dead Schreibers’s long-fingered bats of the *Miniopterus schreibersii* species, EBOV (except for the Reston virus or RESTV) and MARV are highly pathogenic in humans.

EBOV causes an acute and serious viral hemorrhagic fever (VHF) disease, which is often fatal if not treated. EBOV first appeared in 1976 in two simultaneous outbreaks, one in what is now known as Nzara in South Sudan and the other in Yambuku in a village near the Ebola River (for which the virus was named after) in the Democratic Republic of Congo (DRC). The most recent and largest EBOV outbreak since its discovery occurred in 2014–2016 and caused a major concern not only for the large scale of the outbreak and high mortality (>28,600 cases and 11,325 deaths) but also for the fact that it was reported for the first time in West Africa [2]. Specifically, the outbreak started in Guinea and then moved across land borders to Sierra Leone and Liberia (Figure 1). The most recent EBOV outbreak occurred in 2021 near the epicenter of the 2014–2016 epidemic in Guinea [3].

**Figure 1.**
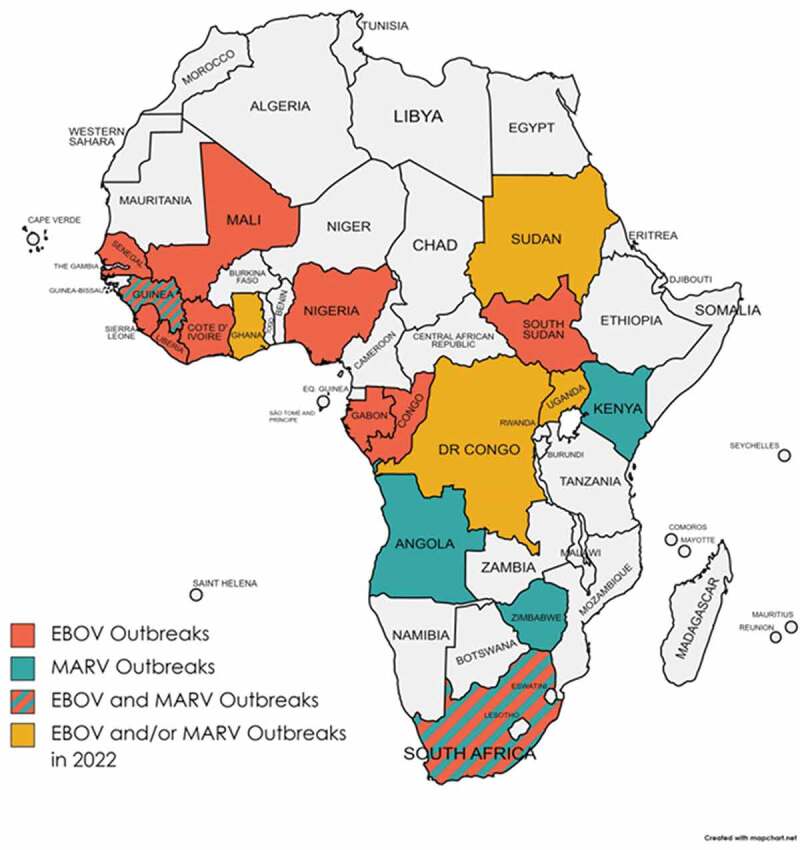
Locations where EBOV and/or MARV outbreaks have occurred are shown. The recently reported EBOV and/or MARV outbreaks (in 2022) are also shown.

Another deadly outbreak caused by a related hemorrhagic fever virus or HF-causing virus (MARV) also occurred in Guinea in 2021 that resulted in 100% case fatality rate [4]. Earlier this year (July 2022), the World Health Organization (WHO) declared yet another MARV outbreak in Ghana [5,6]. These recent HFV outbreaks are significant and notable as many previously known MARV and EBOV outbreaks occurred in other parts of Africa, such as Angola, Kenya, DRC, Uganda, Zimbabwe, South Africa, etc. (Figure 1). For example, since April 2022, there have been three known EBOV outbreaks that occurred in the DRC and Uganda [7].

While EBOV and MARV are often associated with VHF disease outbreaks in Africa, outbreaks caused by MARV have been reported elsewhere outside of the African continent. As a matter of fact, the first reported MARV outbreak happened in Germany in the 1960s. The name of the virus (MARV) came from a German town called Marburg, where the first disease outbreak occurred [8]. Other MARV outbreaks occurred in the capital city of Serbia (Belgrade), where laboratory workers were infected while working with African green monkeys that were imported from Uganda [9]. Fatal cases of MARV infections had also been reported in the United States (US) and the Netherlands in 2008 when the Western travelers had reportedly visited a cave in western Uganda, where bats colonized [10–12]. The transmission of these viruses is believed to occur primarily through direct contact with either the natural animal reservoirs (e.g., fruit bats) or their excreta [1]. Secondary transmissions can occur through contact with blood or bodily fluids of the infected individuals (humans or animals) or with contaminated materials (e.g., soiled clothing and bedding, etc.), or in the hospital setting via either improper use of personal protective equipment/gear (PPE) or contaminated medical instruments or devices.

Recent cases of EBOV and MARV outbreaks in West African countries are alarming partly because people living in those countries are already prone to being infected by another deadly HF-causing virus known as Lassa virus (LASV) [13]. This has greatly complicated the differential diagnosis for these VHF diseases, as they usually present with similar disease signs and symptoms. LASV belongs to the *Arenaviridae* family, in the genera *Mammarenaviridae* of the order *Bunyavirales* that includes other deadly HF-causing viruses, such as Rift Valley fever virus, Crimean-Congo hemorrhagic fever virus, and hantaviruses [14]. LASV was first discovered in 1969 in a town called Lassa (Nigeria) where two missionary nurses were fatally infected [15]. It can cause frequent VHF disease outbreaks in West Africa with recent outbreaks occurring in January 2022 in Nigeria [16] and in April 2022 in Guinea [17]. Although LASV is endemic to West African countries (e.g., Nigeria, Guinea, Sierra Leon, Liberia, etc.) (Figure 1), like MARV and EBOV, traveler-associated cases of LASV infections have been reported in the US and Europe [for a review, see [18]]. This is partly due to the convenience of modern travels across borders and continents.

While the origin for the recent MARV outbreaks (and to some extent EBOV outbreaks) in West Africa in the recent years remains largely unknown, the simultaneous emergence of these viruses amidst the LASV endemicity in several West African countries raises enormous public health concerns as these deadly HF-causing viruses can spread beyond the borders of these countries and into other parts of the world, if they are left unchecked.

Recent Nigeria Centre for Disease Control (NCDC) weekly epidemiological reports [19] describe increasing case numbers of different fatal and life-threatening infectious diseases with pandemic potentials, such as Lassa fever, avian influenza, rubella, measles, malaria, cholera, and more recently, COVID-19 and monkeypox (MPX), that can place a tremendous strain on the already fragile public health and health-care systems of this country. The country’s ability to effectively manage infectious disease outbreaks is currently suboptimal, partly due to the insufficient resources necessary to properly perform disease surveillance and diagnosis in order to inform effective infection prevention and control measures. The relatively porous nature of the borders between Nigeria and neighboring countries in West Africa allows for frequent, uncontrolled, and unchecked cross-border movements of people and their livestock as well as wildlife species that can facilitate the spread of infectious diseases, such as MARV, EBOV, LASV, COVID-19, MPX, among others.

To mitigate the rapid spread of these and perhaps other yet unknown deadly infectious diseases, the One Health approach should be implemented across the animal-human-environment interface. Local, national, and international collaborations must be fostered to enhance early detection and preparedness to tackle disease outbreaks in a concerted, coordinated, and timely fashion. This will require a global awareness of and attention to emerging and re-emerging infectious diseases, enhance training and/or retraining of frontline health-care workers, and modernize health-care infrastructures, communications, and other logistical assets. Accurate medical information about infectious diseases affecting the local communities must be disseminated widely and across various social media platforms in order to combat any intentional or unintentional misconceptions and stigmatizations associated with the disease that is impacting those communities. These grassroot efforts are necessary to avert any potential offensive media content that can go “viral” and can lead to (at best) unhealthy and (at worst) deadly consequences. Meanwhile, transparent (open) and meaningful international collaborations among the scientific and health-care communities and governmental agencies are vital to halt the spread of unanticipated infectious diseases across the international borders. New and sustained levels of international collaborations and supports are also necessary to encourage the development of new and/or improved preventative and therapeutic strategies to mitigate and treat infectious diseases for which no treatment modalities are currently available.

## Data Availability

No primary data are included in this article.
